# Supplementary material for: Development and validation of a clinical prediction model for in-hospital heart failure risk following PCI in patients with coronary artery disease
Source: PLoS One. 2025 Jun 24;20(6):e0325036. doi: 10.1371/journal.pone.0325036 (PMC12186926; doi:10.1371/journal.pone.0325036)
Supplement: S1 Table — (DOCX) [file pone.0325036.s001.docx]

**Table S1. Normal value range of laboratory indicators.**

| Indicators | Normal value range |
| --- | --- |
| HbA1c | 4.0-6.5 (%) |
| Cholesterol | 0-5.18 (mmol/L) |
| Triglyceride | 0-1.71(mmol/L) |
| HDL | 1.03-1.55(mmol/L) |
| LDL | 0-3.37(mmol/L) |
| Urea | 2.6-7.5(mmol/L) |
| Creatinine | 41-73(mmol/L) |
| EF | 50-70(%) |
| LVED | 35-50(mm) |
| cTnI | 0-15.6(pg/ml) |
| MYO | 0-100.2(ng/ml) |
| CKMB | 0-5.8697(ng/ml) |
| NT-proBNP | 0-726(pg/ml) |
| D2 | 0-0.5(ug/ml) |
