# Supplementary material for: Development and validation of a clinical prediction model for in-hospital heart failure risk following PCI in patients with coronary artery disease
Source: PLoS One. 2025 Jun 24;20(6):e0325036. doi: 10.1371/journal.pone.0325036 (PMC12186926; doi:10.1371/journal.pone.0325036)
Supplement: S2 Table — (DOCX) [file pone.0325036.s002.docx]

**Table S2. Regression Coefficients from Multivariable Logistic Analysis of HF Risk After PCI in ACS Patients.**

| Variables | Lasso regression | | | After model simplification | | |
| --- | --- | --- | --- | --- | --- | --- |
|  | *β* | *SE* | *P* | *β* | *SE* | *P* |
| NYHA | 2.219 | 0.361 | <0.001 | 2.223 | 0.346 | <0.001 |
| Smoking | 0.946 | 0.421 | 0.025 | 1.004 | 0.414 | 0.015 |
| RCA occlusion post PCI | 1.007 | 0.457 | 0.028 | 0.974 | 0.445 | 0.029 |
| LVEF | 1.154 | 0.639 | 0.071 | 1.266 | 0.635 | 0.046 |
| NT-proBNP | 1.295 | 0.422 | 0.002 | 1.363 | 0.414 | <0.001 |
| HDL | 0.545 | 0.436 | 0.211 | - | - | - |
| MYO | 0.517 | 0.402 | 0.198 | - | - | - |
| Note: *β*, regression coefficients; *SE*, standard error; HDL, high-density lipoprotein; RCA, right coronary artery; LVEF, left ventricular ejection fraction; MYO, myoglobin; NYHA, New York Heart Association classification; PCI, Percutaneous coronary intervention. | | | | | | |
