# Supplementary material for: Development and validation of a clinical prediction model for in-hospital heart failure risk following PCI in patients with coronary artery disease
Source: PLoS One. 2025 Jun 24;20(6):e0325036. doi: 10.1371/journal.pone.0325036 (PMC12186926; doi:10.1371/journal.pone.0325036)
Supplement: S3 Table — (DOCX) [file pone.0325036.s003.docx]

**Table S3.** **Multivariable logistic regression analysis stratified by gender and age**

| Subgroup | Variables | Final model | | | | |
| --- | --- | --- | --- | --- | --- | --- |
|  |  | *β* | *SE* | *OR* | 95% *CI* | *P* |
| Male  (n = 247) | NYHA | 2.255 | 0.448 | 9.533 | 4.317- 24.680 | **<0.001** |
|  | Smoking | 1.226 | 0.477 | 3.406 | 1.373 - 9.070 | **0.010** |
|  | RCA occlusion post PCI | 1.149 | 0.515 | 3.154 | 1.136 - 8.727 | **0.026** |
|  | LVEF | 1.506 | 0.762 | 4.507 | 0.979 - 19.984 | **0.048** |
|  | NT-proBNP | 1.397 | 0.490 | 4.042 | 1.534 - 10.643 | **0.004** |
| Female  (n = 62) | NYHA | 1.961 | 0.549 | 7.107 | 2.872 - 26.373 | **<0.001** |
|  | Smoking | -15.771 | 2203.232 | - | - | 0.994 |
|  | RCA occlusion post PCI | 0.707 | 1.049 | 2.027 | 0.236 - 16.048 | 0.500 |
|  | LVEF | 1.118 | 1.405 | 3.058 | 0.158 - 48.209 | 0.426 |
|  | NT-proBNP | 1.228 | 0.916 | 3.415 | 0.600 - 24.528 | 0.180 |
| ≤ 58 years old  (n = 155) | NYHA | 3.414 | 0.775 | 30.372 | 7.828 - 170.246 | **<0.001** |
|  | Smoking | 0.878 | 0.629 | 2.406 | 0.709 - 8.834 | 0.163 |
|  | RCA occlusion post PCI | 0.887 | 0.709 | 2.428 | 0.558 - 9.459 | 0.211 |
|  | LVEF | -0.485 | 1.567 | 0.616 | 0.025 - 9.276 | 0.757 |
|  | NT-proBNP | 0.347 | 0.753 | 1.415 | 0.272 - 5.672 | 0.645 |
| > 58 years old  (n = 154) | NYHA | 1.916 | 0.414 | 6.792 | 3.286 - 16.832 | **<0.001** |
|  | Smoking | 1.444 | 0.622 | 4.237 | 1.288 - 15.191 | **0.020** |
|  | RCA occlusion post PCI | 0.937 | 0.619 | 2.551 | 0.756 - 8.785 | 0.130 |
|  | LVEF | 2.093 | 0.815 | 8.107 | 1.687 - 42.867 | **0.010** |
|  | NT-proBNP | 1.987 | 0.596 | 7.292 | 2.370 – 25.326 | **<0.001** |
| Note: *β*, regression coefficients; *SE*, standard error; *CI*, confidence interval; RCA, right coronary artery; LVEF, left ventricular ejection fraction; NYHA, New York Heart Association classification; *OR*, odds ratio; PCI, Percutaneous coronary intervention. | | | | | | |
