# Supplementary material for: Development and validation of a clinical prediction model for in-hospital heart failure risk following PCI in patients with coronary artery disease
Source: PLoS One. 2025 Jun 24;20(6):e0325036. doi: 10.1371/journal.pone.0325036 (PMC12186926; doi:10.1371/journal.pone.0325036)
Supplement: Attachment — Submitted filename: Respond to Reviewer.docx minor revision.docx [file pone.0325036.s007.docx]

**Dear Editor and Reviewers,**

Thank you for your constructive feedback and the opportunity to revise our manuscript. We sincerely appreciate the time and expertise invested by the reviewers, particularly Reviewer #1’s detailed comments, which have significantly strengthened the methodological rigor and clarity of our work.

**Key Improvements in the Revised Manuscript:**

All concerns raised by Reviewer #1 have been thoroughly addressed, with methodological rigor enhanced and discussions expanded.

Reviewer #2 had no further concerns, confirming the validity of our findings.

Detailed point-by-point responses to each comment are provided in the attached file. The revised manuscript and supplementary materials are also uploaded for your convenience.

We hope these revisions meet the journal’s standards and welcome any further suggestions to improve the manuscript.

Yours sincerely,
Bin Huang

**Respond to Reviewer #1:**

Major Weaknesses:

Sample Size and Population Bias: The small sample (n=309), with nearly 80% male participants, limits statistical power and generalizability. While the limitation is acknowledged, the authors should provide further analysis stratified by gender and discuss implications more explicitly.
Exclusion of Important Predictors: Key clinical predictors such as lesion length, peak troponin, inflammatory markers, and number of stents were omitted. The reasons provided are understandable, but their clinical impact on prediction performance must be critically discussed, particularly for lesion severity and troponin as established HF predictors.
Absence of Comparator Risk Scores: No comparison was made with widely used scoring systems such as GRACE, TIMI, Killip, or SYNTAX scores. Reviewer #1 clearly requested this. Without benchmarking against these models, it is difficult to judge the added value of the proposed tool.
Incomplete Outcome Definition: The criteria used to diagnose in-hospital HF post-PCI, especially whether standardized guidelines or clinical judgment were used, remain insufficiently detailed.

Detailed Comments:

1.The cited literature on coronary revascularization and LV dysfunction is relevant. However, the rationale for developing a new model—rather than modifying existing scores—needs better justification.

**Response:** Thank you for the comment. We have revised the introduction to better clarify the aim of our study. The model developed by Yu et al. focused on predicting in-hospital mortality in patients who had already developed heart failure after PCI. In contrast, our model is designed to predict the occurrence of new-onset heart failure during hospitalization following PCI. Our model aims to help identify high-risk patients early and support timely clinical intervention (Lines 56–61).

2.Provide a clearer operational definition of heart failure diagnosis.

**Response:** In response to your valuable feedback regarding potential gaps in diagnostic criteria, we have updated and refined the heart failure diagnostic framework by synthesizing evidence from the 2021 ESC and 2022 ACC/AHA consensus guidelines. The revised criteria systematically incorporate clinical manifestations (symptoms and signs), objective biomarkers, imaging parameters, and exclusionary diagnoses, while establishing a dynamic assessment process that integrates symptom evaluation, physical examination findings, biomarker analysis, and imaging studies (Lines 100–114).

3. Specify how missing data were managed and whether any variable interactions were explored.

**Response:** We are very grateful to the reviewers for their valuable comments. We generated five imputed datasets through 100 iterations of the multivariate imputation by chained equations (MICE) method (Lines 183–185).

4 Include a rationale for excluding key variables (e.g., lesion length, troponin) in the Methods section.

**Response:** Thank you very much for your valuable comments. A large amount of missing data could likely introduce biases in the analysis. Thus, to ensure the reliability of the results, we decided to exclude them (Lines 149–152).

5. Provide stratified model performance by gender or age to assess potential bias.

**Response:** Thank you for your instruction. We've addressed the need to provide stratified model performance by gender and age. Table S3 in the manuscript shows the model results across these subgroups (Lines 1623–165, Lines 211–215).

6. Include the full regression coefficients in supplementary materials.

**Response:** Thanks for the instruction. We've added the full regression coefficients to the supplementary materials as required. You can find them in Table S3.

7. Expand on how the model compares (or could compare) with existing models.

**Response:** We sincerely accept your suggestion of the need for model comparison. In the original manuscript, we mainly described the similarities between our model and existing ones. Following your advice, we have now added a more detailed comparison of the differences in predictor variables, which may provide readers with a more comprehensive understanding of our model’s unique contributions (Lines 392–410).

8. Critically evaluate the omission of key clinical variables and scoring systems.
**Response:** Thank you for your insightful comment on the omission of key variables and scoring systems. In our initial study, due to extensive missing data, we overlooked crucial variables like lesion length. The absence of widely-used scores, such as the Syntax score, also limited our study. In the revised manuscript, we have objectively evaluated the implications of these omissions and highlighted our study's limitations (Lines 355–371).

9. Strengthen the rationale for including each of the five final predictors.
**Response:** Thank you for your careful review and the valuable comment. We selected the five predictors by considering both statistical significance and clinical applicability. These variables are easy to obtain in practice and may enhance the model’s usefulness in real-world clinical application. The relevant reasons have been elaborated in the article (Lines 254–264).

10. Clarify how RCA occlusion impacts right heart failure risk based on literature.

**Response:** I truly appreciate your astute question. When the RCA is occluded, it directly disrupts the blood supply to the right ventricle. This disruption can lead to reduced oxygen and nutrient delivery, subsequently weakening the right ventricular function and significantly increasing the susceptibility to right heart failure. In the revised manuscript, we have elaborated it (Lines 301–334).

11. The authors should provide further analysis stratified by gender and discuss implications more explicitly.

**Response:** Thank you for the suggestion. We have added subgroup analyses by gender and age in the revised manuscript. The model performed well in males and older patients, while performance was limited in females and younger patients, likely due to sample size imbalance. We also discussed the implications and supported the need for future models tailored to different subgroups (Lines 335–354).

12. Temper statements about clinical application and emphasize the need for external validation and model refinement.

**Response:** We sincerely appreciate your suggestions. We have emphasized the importance of external validation and the need for further model refinement in the revised manuscript. In addition, we have revised the overall conclusion of the study to be more cautious, reflecting the preliminary nature of the findings. (Lines 471–475).
